# A quick test of cognitive speed can predict development of dementia in Parkinson’s disease

**DOI:** 10.1038/s41598-019-51505-1

**Published:** 2019-10-28

**Authors:** Mattis Jalakas, Sebastian Palmqvist, Sara Hall, Daniel Svärd, Olof Lindberg, Joana B. Pereira, Danielle van Westen, Oskar Hansson

**Affiliations:** 1 Clinical Memory Research Unit, Department of Clinical Sciences, Malmö, Lund University, Lund, Sweden; 2 Department of Neurosurgery, Skåne University Hospital, Skåne, Sweden; 3 Department of Neurology, Skåne University Hospital, Skåne, Sweden; 4 Memory Clinic, Skåne University Hospital, Skåne, Sweden; 5 Diagnostic Radiology, Lund University, Lund, Sweden; 6 Medical Imaging and Physiology, Skåne University Hospital, Skåne, Sweden; 7 Division of Clinical Geriatrics, Department of Neurobiology, Care Sciences and Society, Karolinska Institute, Stockholm, Sweden

**Keywords:** Neuroscience, Brain

## Abstract

Parkinson’s disease (PD) patients frequently develop cognitive impairment. There is a need for brief clinical assessments identifying PD patients at high risk of progressing to dementia. In this study, we look into predicting dementia in PD and underlying structural and functional correlates to cognitive decline in PD. We included 175 patients with PD, 30 with PD dementia, 51 neurologically healthy controls and 121 patients with Alzheimer’s disease (AD) from Skane University Hospital, BIOFINDER cohorts. All underwent cognitive tests, including MMSE, 10-word list delayed recall (ADAS-cog), A Quick Test of cognitive speed (AQT), Letter S fluency, Clock Drawing Test (CDT) and pentagon copying. In non-demented patients with PD, abnormal AQT and CDT results predicted an increased risk of subsequent development of dementia (hazard ratio 2.2 for both). When comparing the cognitive profile between PD and AD, decreased performance on AQT, which measures attention and processing speed, was more typical in PD. Lastly, we investigated the underlying structural and functional correlates for the PD-specific test AQT with magnetic resonance imaging. In PD patients, decreased performance on AQT was associated with i) cortical thinning in temporoparietal regions, ii) changes in diffusion MRI, especially in the cingulum tract, and iii) decreased functional connectivity in posterior brain networks.

## Introduction

Parkinson´s disease (PD) is the second most common neurodegenerative disease^1^ and a common cause of motor symptoms, cognitive decline and dementia. The overall prevalence of PD is approximately 1% of the population >60 years of age. It has been estimated that 75% of patients with PD that survive more than 10 years develop dementia with a point prevalence of close to 30%^2^, although some studies suggest a lower cumulative prevalence of 46%^3^. The development of dementia is a serious problem that severely worsen the quality of life of patients and their relatives^4^ and increases the costs for society^5^.

Accurately predicting and diagnosing PD with dementia (PDD) is not only important for allocating necessary resources and care-giver support^6^, but also for treating the patients correctly. Randomized clinical trials (RCT) have shown positive cognitive effects of acetylcholinesterase inhibitors^7^ and memantine^8^ in PDD. Prediction of PDD may in the future also be valuable for choosing and evaluating neuromodulatory treatments.

Previously, memory and executive impairment has been shown to predict dementia in PD^9^ as well as impaired pentagon copying^10^. However, perhaps due to underlying heterogeneous pathologies, predicting dementia in PD remains a challenge, and the underlying pathology needs to be more elucidated.

The three aims of this study were to (1) evaluate the performance of various brief cognitive tests for predicting subsequent development of PD dementia, (2) identify tests specific to the cognitive decline in PD, and (3) examine the underlying brain correlates of the cognitive deficits in PD. First, we studied which cognitive tests could predict development of dementia in patients with PD (n = 175) during a mean of 5.5 (SD 2.3) years of follow-up. Next, patients with PDD at baseline (n = 30) were compared to an independent cohort of AD patients (n = 121) to evaluate which tests were specific for PDD.

Finally, in an attempt to shed light on specific patterns of pathology in PD dementia. we evaluated the underlying functional and structural correlates of declining processing speed in PD with magnetic resonance imaging (MRI) modalities, evaluating cortical thickness, white matter tracts and functional connectivity.

## Methods

### Participants

All participants were recruited at Skane University Hospital in southern Sweden and included patients with PD, PDD, AD as well as neurologically healthy controls. The cases are part of the prospective and longitudinal Swedish BioFINDER study (Bio*markers For Identifying Neurodegenerative Disorders Early and Reliably*) described in previous publications from our group^11^ and at http://biofinder.se. This study started in 2008 and is still ongoing. The PD patients met the National Institute of Neurological Disorders and Stroke Diagnostics Criteria for PD^12^. The patients with PDD met the clinical diagnostic criteria for dementia associated with PD according to Emre *et al*.^13^. The healthy controls (HC) served as a control group and were neurologically and cognitively healthy. The AD patients met the Diagnostic and Statistical Manual of Mental Disorders (Third Edition Revised) criteria for dementia and the criteria for probable AD defined by the National Institute of Neurological and Communicative Disorders and Stroke-Alzheimer´s and Related Disorders Society^14^.

### Clinical evaluation

The study participants underwent cognitive evaluation according to the proposed algorithm and instruments for establishing the diagnosis of PDD at Level I by Dubois *et al*.^15^ and the DSM-V criteria^16^. The evaluation was performed at baseline and 2^nd^ year for up to 10 years (follow-up range: 1.5–10 years). Conversion to dementia was evaluated at every time point (2, 4, 6, 8 or 10 year), or by chart review in the case of dropouts. A physician with experience in movement disorders performed a detailed history and neurological exam at every visit, as well as a review of the patient’s charts. A research nurse performed the cognitive testing. In this study, MMSE was chosen as the measure of global cognitive function. To measure episodic memory, the 10-word list delayed recall from the Alzheimer´s Disease Assessment Scale – cognition (ADAS-cog) was used^17^. Phonemic fluency was measured with Letter S fluency^18^ and visuospatial function with the Clock Drawing Test (Shulman’s scoring method^19^) and pentagon copying^20^, scored using the scoring method suggested by Caffara *et al*.^21^. Attention and processing speed was measured with AQT^22–24^. Briefly, AQT is a time trial of naming forms, colours and lastly the combination of coloured geometrical figures. The test aims to measure processing speed and executive function. It is a time trial where the figures are described by form and colour first separately, then in combination. The measured variable is the time it takes to name of 40 colors/forms. Motor function was assessed using UPDRS-III^25^. All patients were examined in the ON-state. For the longitudinal analysis of clinical progression in the PD cohort, all PD patients (n = 175) that were non-demented at baseline were followed clinically for up to ten years, (mean 5.3, SD 2.3, range 1.5–10). A physician with experience in neurodegenerative disorders evaluated the patients for presence of PDD at baseline or conversion to PDD at every follow up visit, based on clinical test scores and global functioning at home and in society. For dropouts, patients’ medical records were also reviewed for information about conversion to PDD during the follow up period. In the longitudinal analysis, a predefined AQT Color Form cutoff of >80 seconds was used (+2 SD above mean in a reference population^26^) and a predefined cutoff of <4 points was used for the Clock Drawing Test^19^.

### MR imaging acquisition and processing

For investigation of regional brain changes associated with cognitive impairment, we performed MR imaging in 108 cases with PD and 17 cases with PDD. The clinical information closest to the MRI scan was analysed. All MR imaging was performed on 3 T Siemens Skyra MR equipment using the standard 20 channel headcoil. Coronal MPRAGE comprised 180 slices, voxel size 1 × 1 × 1 mm^3^. The resting state-fMRI protocol comprised 256 T2*-weighted echo planar imaging volumes sensitized to the BOLD effect (echo time 30 ms; repetition time 1850 ms; 33 axial slices; matrix 64 × 64; voxel size 3 × 3 × 3.75 mm^3^). Subjects were instructed to lie still with their eyes closed, without thinking of anything in particular and without falling asleep. DKI data comprised 99 volumes with 52 continuous slices, three volumes with b = 0 s/mm^2^ and 96 volumes with b-values of 250, 500, 1000, and 2750 s/mm^2^, distributed over 6, 6, 20, and 64 directions^27^. The voxel size was 2.3 × 2.3 × 2.3 mm^3^ and FOV 294 × 294 × 120 mm^3^. Motion and eddy current distortions were corrected using an extrapolation-based method for improved high b-value performance. Image volumes were smoothed using an isotropic 3D Gaussian kernel with a full-width at half maximum of 2.3 mm^28–30^. DKI parameter maps of fractional anisotropy (FA), mean kurtosis (MK), and mean diffusivity (MD)^31^ were obtained using in-house developed software which fitted the diffusion and kurtosis tensors by nonlinear optimization.

### Freesurfer analysis

Using the free image analysing tool Freesurfer, available at http://surfer.nmr.mgh.harvard.edu, we analysed T1MPRAGE MR images.

Freesurfer software package is a validated tool for measuring cortical thickness^32^. Non-brain tissue is removed and grey and white matter is separated. The post processing results were quality controlled with visual inspection prior to analysis. Three patients were excluded due to corrupted data. Using the QDEC application in the free Freesurfer software package, we correlated the AQT scores, as well as the results from the other cognitive tests (MMSE, CDT, Pentagon copying, Letter S fluency and Delayed Word recall), to cortical thickness as the dependent variable in a whole brain analysis, using Monte Carlo simulation of 1.3. The model was balanced for age and years of education.

### DTI/tractography

Tractography using deterministic tracking based on constrained spherical deconvolution (CSD) was performed to generate the left and right dorsal cingulum (CG), the hippocampal CG, the corticospinal tract (CST) the fornix (FX), the uncinate fasciculus (UF), the superior longitudinal fasciculus (SLF), the inferior longitudinal fasciculus (ILF), and the inferior fronto-occipital fasciculus (IFO)^33^. One seed-ROI covering the full extent of the specific tract and logical AND- and NOT-ROIs were defined in Montreal Neurological Institute (MNI) 152 standard-space and warped back to native-space utilizing the warp-fields generated by FLIRT and FNIRT^34^. All tracts were visually inspected and ROIs were adjusted in order to secure location of the tract in the intended anatomical region with the number of streamlines below 100. The average parameter estimate for each tract was extracted and used in the subsequent analyses. Since the differences we aimed to elucidate could be relatively subtle, we abstained from a whole brain approach WBA, because of the risk of it being to insensitive considering the multiple comparison issues with such voxel-based approach.

### Functional network analysis

fMRI scans were preprocessed using the Statistical Parametric Mapping (SPM8) software (http://www.fil.ion.ucl.ac.uk/spm). In brief, the following steps were applied: removal of first six volumes, correction for time offsets between slices, realignment, normalization to the MNI template, temporal high-pass filtering (0.01–0.08 Hz), regression of six head motion parameters, and white matter and cerebrospinal fluid signals. To remove the influence of volumes affected by motion, we applied a scrubbing procedure using root-mean-square intensity differences between volumes N to volumes N + 1 with DVARS^35^. In this study, 1 PD and 2 PDD patients showed head motion > =3 mm translation and rotation, and for this reason they were excluded from the analyses.

Functional brain networks were constructed for each subject using a set of nodes representing brain regions. These nodes were connected by edges representing the statistical interdependence in blood oxygen level-dependent (BOLD) signals. The nodes were defined using the 200 cortical and subcortical regions provided by the Craddock atlas^36^. The edges were calculated as the Pearson correlation coefficients between the regional time series of all possible pairs of regions, resulting in a 200 × 200 correlation matrix for each subject. To assess whether functional connectivity between the previous brain regions was associated with AQT performance, we used the network-based statistic (NBS) software package^37^.

### Statistical analysis

SPSS 25 was used for all statistical analysis except for the Freesurfer data where the built in application Query Estimated Design Contrast, QDEC, was used^38^. For comparisons between the different diagnostic groups at baseline (PD, PDD, and HC), a univariate linear model corrected for age, sex, years of education and disease duration was applied with corrections for multiple comparisons using the Bonferroni method. The PD and AD patients were compared with a similar model, corrected for MMSE and age as a measure of global cognitive function and disease burden. For correlation analysis between cognitive tests and tractography, partial Pearson correlations were performed, corrected for age, sex, years of education, disease duration and brain volume. Since these analyses were exploratory in nature, we did not correct for multiple comparisons. For evaluation of the predictive value of cognitive tests on subsequent development of dementia, we performed cox regression analysis corrected for age, sex, disease duration and years of education.

In Freesurfer, the built-in application QDEC was used for analysis. The analysis was adjusted for age, sex, years of education and disease duration. A Monte Carlo simulation was used and p < 0.05 was considered significant. For the functional network analysis, a regression analysis was carried out to test whether AQT scores correlated with functional connectivity in the whole patient group, while controlling for age, sex, education and disease duration. Correction for multiple comparisons was performed using a false discovery rate (FDR) of 0.001 (1000 permutations).

### Ethics

All individuals gave their written informed consent. The study procedure was approved by the local ethics committee at Lund University, Sweden, and conducted according to the Helsinki Declaration.

## Results

### Baseline characteristics

The baseline demographics of the participants are presented in Table 1. Group comparisons revealed differences in letter S fluency and pentagon drawing between HC and PD, but the differences were relatively small. The PDD patients performed worse on all cognitive scores except CDT than the PD and HC patients.

**Table 1:** Baseline demographics.

	HC (n = 51)	PD (baseline) (n = 175)	PDD (n = 30)
Baseline age, years	65 (8.5)	65 (10)	72.5 (6.6)
Gender (1 = female)	0.56	0.37	0.3
Years of education	13.3 (3.2)	13 (4.0)	11 (4.5)
Disease Duration Years		5.1 (4.9)	14 (6.8)
Levodopa equivalents		455 (455)	881 (549)
UPDRS-III	1.7 (2.6)	16 (10)^c^	35 (13)^f^
MMSE (0–30 points)	28.3 (1.5)	28.5 (1.3)	22 (5.0)^f i^
Wordlist delayed recall	2 (1.7)	3 (2.1)	6 (3.0)^f i^
**(0–10 errors)**			
AQT Color Form (sec)	63 (11)	71 (29)	181 (104)^f i^
AQT Color (sec)	24 (4.5)	27 (7.0)	52 (30)^f i^
AQT Form (sec)	35 (7.5)	37 (9.3)	75 (37)^f i^
Letter S fluency	17.5 (5.9)	14.7 (5.7)^a^	7.9 (4.1)^f i^
Clock Drawing Test (0–5p)	4.1 (0.7)	4.2 (0.9)	4.6 (0.6)
Pentagon Drawing (0–13p)	12 (0.9)	11.3 (1.3)^b^	7.5 (4.0)^f i^

Mean presented with standard deviations within parenthesis.

To adjust for multiple comparisons with Bonferroni method, all p-values were multiplied by 3. All comparisons were corrected for age, gender, years of education and disease duration.

^a^Significant difference (p < 0.05), comparison between HC and PD.

^b^Significant difference (p < 0.01), comparison between HC and PD.

^c^Significant difference (p < 0.001), comparison between HC and PD.

^d^Significant difference (p < 0.05), comparison between HC and PDD.

^e^Significant difference (p < 0.01), comparison between HC and PDD.

^f^Significant difference (p < 0.001), comparison between HC and PDD.

^g^Significant difference (p < 0.05), comparison between PD and PDD.

^h^Significant difference (p < 0.01), comparison between PD and PDD.

^i^Significant difference (p < 0.001), comparison between PD and PDD.

### Prediction of PDD conversion

Next, we studied which cognitive tests could predict subsequent development of dementia in non-demented PD patients. The patients were followed between 1.5 and 10 years with an average of 5.5 (SD 2.3) years. In total 41 (23%) PD patients converted to dementia during follow-up. We applied a Cox regression model corrected for age, sex, disease duration and years of education. Z-scores of the results on the cognitive tests were used for comparability and the results are presented in Table 2. In brief, AQT Form, AQT Color Form and the CDT at baseline indicated an increase hazard ratio (HR) of developing dementia during follow up. Further, the significant predictors (AQT and CDT) were dichotomised into normal and abnormal scores using the predefined cutoffs CDT < 4 points and AQT Color Form >80 seconds. Using these cutoffs, AQT Color Form predicted dementia development with an hazard ratio (HR) of 2.2 (95% CI 1.08–4.5) and the CDT had a similar HR of 2.2 (95% CI 1.08–4.6).

**Table 2:** Single test risk stratification of PD dementia conversion.

Cognitive test	HR	p-value	95% CI
MMSE	1.2	0.39	0.80–1.8
AQT Color Form	1.6	**0.04**	1.01–2.7
AQT Color	1.2	0.2	0.9–1-0.6
AQT Form	1.6	**0.001**	1.2–2.0
10-world list delayed recall	1.3	0.12	0.9–1.9
Letter S fluency	0.7	0.13	0.48–1.1
Months backwards	1.1	0.5	0.79–1.6
Pentagon drawing	0.56	0.55	0.314–1.01
Clock Drawing Test	1.4	**0.032**	1.03–2.0

COX regression evaluating the z-scores of the cognitive tests as predictors for conversion to dementia in non-demented PD patients. Each cognitive test model was adjusted for age, sex, years of education and disease duration. HRs are shown based on test z-scores for comparison between tests.

### Comparison of AQT and the clock drawing Test in PDD and AD

Next, we compared the cognitive profile of our PDD patients to that of an independent AD cohort to evaluate if any of the two predictive tests with very similar predictive power (AQT and Clock Drawing Test) were more specific to PD. The model was corrected for age and MMSE as proxy of disease severity. We found that the PD patients exhibited slower processing speed on all AQT tasks whereas there was no difference on the Clock Drawing Test (Table 3). As expected, the AD patients performed worse on the memory task (word list delayed recall).

**Table 3:** Comparison of cognitive profile between AD and PDD patients.

	AD (n = 121)	PDD (n = 30)	P-value
Baseline age, years	77 (5.5)	72 (6.0)	<0.001
Years of education	10 (3.2)	11 (4.5)	0.143
MMSE	22 (4.0)	22 (5.0)	0.868
Wordlist delayed recall (0–10 errors)	8.5 (1.6)	6.0 (2.9)	<0.001
AQT Color Form (sec)	108 (35)	175 (98)	<0.001
AQT Color (sec)	38 (14)	52 (20)	<0.001
AQT Form (sec)	60 (25)	74 (37)	0.003
Clock Drawing Test (0–5p)	3.1 (1.3)	3.1 (10.9)	0.61

Data are shown as mean values with standard deviations within parenthesis.

Cognitive test comparisons were adjusted for age, years of education and global cognitive function (MMSE).

### Associations between AQT and MR imaging

Since our results suggested that AQT not only predicted conversion to PDD in PD but also seems more typical to PD than AD, we investigated the underlying structural and functional correlates to this seemingly PD specific test in an attempt to elucidate underlying pathological patterns. This was done by analysing associations between processing speed (measured with AQT Color Form) and cortical thickness, white matter tracts and functional networks in patients with PD or PDD who underwent MRI (n = 125). Clinical data for this subset of the cohort is presented in a supplement (Supplementary Table 1).

Analysis of all available cortical thickness measures adjusted for age and years of education, revealed that reduced performance on AQT was significantly correlated with cortical thinning in the entorhinal, inferior parietal and inferior temporal cortex in the left hemisphere and the lateroorbitofrontal cortex in the right hemisphere (Table 4).

**Table 4:** Correlations between AQT and cortical thickness.

Regions left side	p-value	Regions right side	p-value
Enthorihnal	<0.05	Lateroorbitofrontal	<0.05
Inferiotemporal	<0.05		
Inferiopearietal	<0.05		

QDEC analysis adjusted for age, years of education and disease duration.

**Figure 1:**
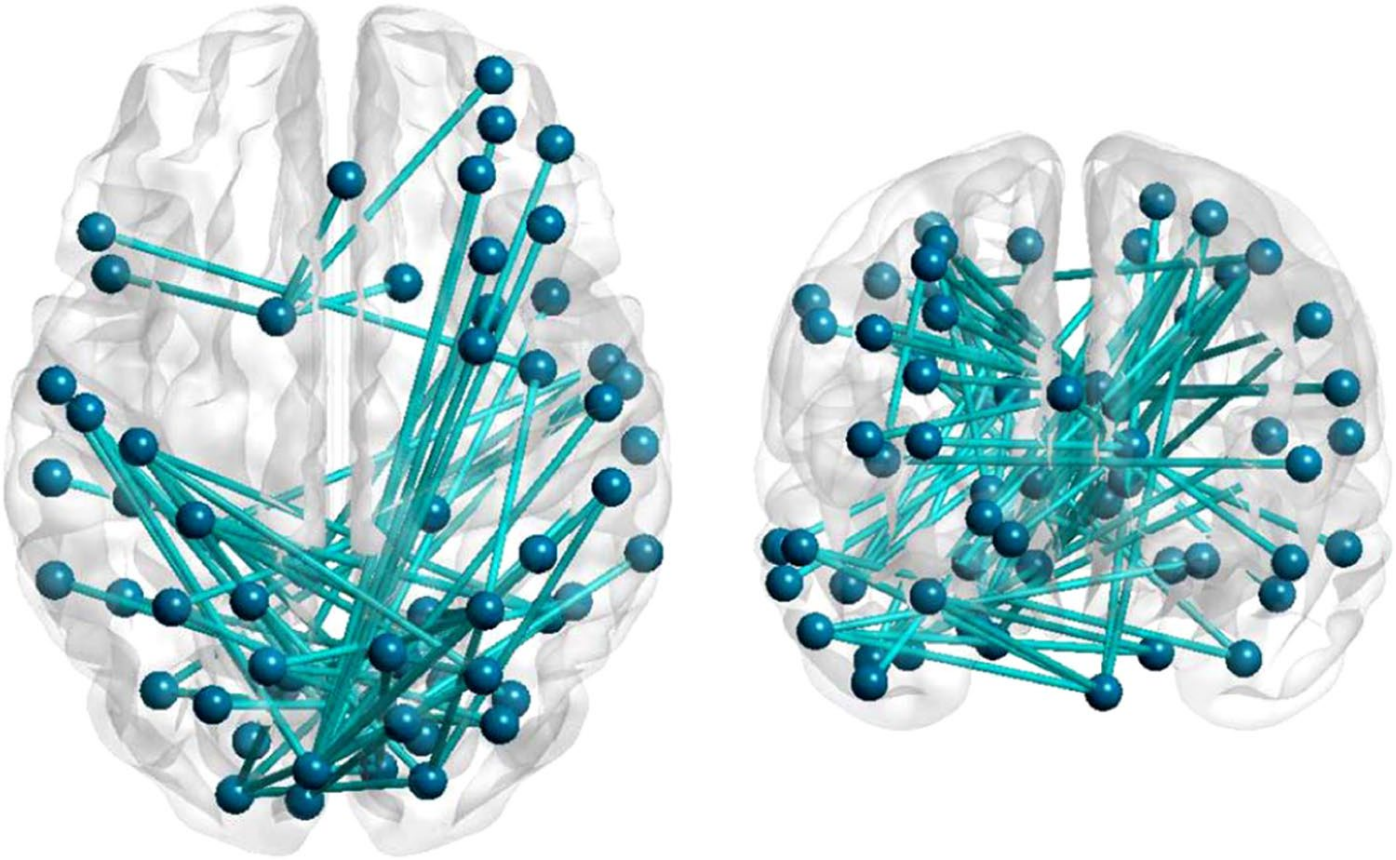
Functional connection network analysis adjusted for age, sex, years of education and disease duration.

Next, white matter tracts were evaluated for correlations with declining processing speed measured with AQT, in an exploratory manner (Table 5). We mainly found correlations between declining processing speed and discrepancies in the cingulum tract using the mean diffusivity, MD, parameter, see Table 4. Anomalies could also be found in the inferior fronto-occipital fasciculus, ILF and inferior longitudinal fasciculus, SLF, whereas the corticospinal tract, included as a reference tract, fornix and uncinate fasciculus were not affected. Finally, we studied the associations between AQT Color Form and connectivity using resting-state functional MRI. We identified a network of 60 regions and 72 connections showing a negative correlation with AQT performance in patients (p < 0.001) (Fig. 1). This network mainly consisted of posterior cortical regions such as the precuneus, superior parietal gyri, occipital gyri, cuneus and lingual gyri. In addition, the brainstem, cerebellum and some frontal and temporal regions also correlated negatively with AQT scores, while controlling for age, sex, education and disease duration.

**Table 5:** Partial correlations between AQT and white-matter tracts corrected for age, sex, disease duration, brain volume and years of education.

Tract	Correlation dx (right)	p-value	Correlation sin (left)	p-value
CD MD	**0.274**	**0.007**	**0.222**	**0.03**
CV MD	**0.274**	**0.007**	0.162	0.133
CST MD	−0.065	0.525	−0.043	0.676
UF MD	0.192	0.059	0.139	0.175
SLF MD	0.093	0.366	0.17	0.096
ILF MD	**0.214**	**0.035**	**0.2**	**0.05**
IFO MD	**0.22**	**0.024**	0.148	0.148
FX MD	0.036	0.728		

CD, Dorsal cingulum; CV, Ventral cingulum; CST, Cortico spinal tract, IFO, Inferior fronto-occipital fasciculus, ILF, inferior longitudinal fasciculus, SLF, Superior longitudinal fasciculus, UF, Uncinate fasciculus, FX fornix, represented in column dx but is not side specific.

## Discussion

In this study, we found that both AQT and the Clock Drawing Test predicted increased risk of developing dementia in PD (HR 2.2). When comparing cognitive profiles between PDD and AD, AQT was more specific to PDD, and we therefore further examined the underlying structural correlates of AQT performance (i.e. declining processing speed) in PD and PDD. We found that poor performance on AQT was associated with cortical atrophy in temporoparietal areas (mainly in the left hemisphere), diffusion changes in certain white matter tracts, mainly the cingulum tract, and decreased connectivity in posterior networks.

A few cognitive tests have previously been shown to predict conversion to dementia in PD. For example, Barker *et al*. found evidence that both verbal fluency, with a relative risk (RR) of 9.4 (95% CI 1.9–47.5) for a global decline, and inaccurate pentagon copying, with a RR of 5.2 (95% CI 1.9–14.1), predicted dementia in PD^39^. This could not be replicated in our cohort (Table 2). In the present study, we instead found that AQT and the Clock Drawing Test predicted dementia in PD, but not MMSE, pentagon copying, verbal fluency or word list delayed recall. The prediction of dementia using AQT is in agreement with previous findings showing that conversion to dementia can be predicted with tests of attention and executive function^9^.

Even though both AQT and Clock Drawing Test predicted subsequent development of dementia, AQT was more specific to the cognitive decline in PDD. When comparing the cognitive profile between PDD and AD, AQT scores were worse in the PDD group, but no difference was seen for Clock Drawing Test scores. As expected, the AD patients performed worse on delayed recall, but delayed recall did not seem to increase the risk of developing dementia in PD, suggesting that decline in episodic memory is not a typical or early feature in PDD. This is in accordance with previous studies showing that the cognitive profile in PD is initially more marked by dysexecutive symptoms and decreasing processing speed^40,41^, whereas AD patients decline more in episodic memory^42,43^, even though multiple cognitive domains can be affected in both disorders^40,43^.

The neuropathology of PDD is complex. Likely, a combination of α-synuclein pathology and AD-like pathology (amyloid-β and tau aggregates) is responsible for the cognitive symptoms^44^. Which pathology is more important is not fully elucidated^45^, but some authors suggest that α-synuclein pathology predominates^46^. It has been suggested that the cognitive symptoms are not solely a result of dopaminergic loss but also cholinergic insufficiency (which is thought to cause attention deficits)^46,47^, as well as degeneration of other neuronal cell types in the cortex. Previous studies of cortical thickness in different stages of PD have shown correlations between disease stage and cortical thinning^48^. For example, Zarei *et al*. found correlations between decreased MMSE and cortical thinning in several cortical areas in PD, including the posterior cingulate and precuneus. In our study, worse AQT scores correlated to decreased thickness in the posterior cingulate cortex as well as in the inferior parietal and temporal areas.

When it comes to structural connectivity, Kamagata *et al*. found decreased FA values in the cingulum tract in PD and PDD^49^. In our study we also found weak correlations between decreased processing speed and increased MD values in the cingulum as well as in the ILF and IFO tracts connecting the occipital lobe to the temporal lobe and frontal lobe, respectively^50^. While other studies have found evidence of frontostriatal involvement in dysexecutive syndromes in PD^51^, we also found associations between AQT and decreased connectivity in posterior networks and tracts with occipital connections. This could represent deficits in rudimentary visual processing and set-shifting, which is in agreement with the brain regions that are involved during the AQT Color Form task as visualized using fMRI and regional cerebral blood flow measures (rCBF)^52^.

A limitation of the study is that a larger battery might have identified other predictive tests for dementia conversion in PD, but, nonetheless, our cognitive battery included all tests recommended by the Movement Disorder Society Task Force^15^ and even some additional ones. The structural MRI findings were considered exploratory in nature, and therefore not corrected for multiple comparisons. Further studies are therefore warranted to confirm these findings. Strengths of the study are the relatively large and well described cohort, the large proportion of the original cohort that underwent MRI, and the long and structured follow-up.

We conclude that the dementia in PD is predominantly characterized by declining processing speed and executive dysfunction, which can be evaluated with the readily available test AQT. AQT can also predict subsequent development of dementia. Using the predefined cutoff of >80 seconds for AQT Color Form, clinicians can identify PD patients who have increased risk of developing dementia. Declining processing speed seems to have structural correlates in the cingulum tract and posterior functional networks. Further studies are needed to develop more precise methods and markers to predict cognitive decline in PD, and to shed more light on the underlying structural basis of cognitive decline in PD.

## Supplementary information

Dataset1
