# Supplementary material for: A quick test of cognitive speed can predict development of dementia in Parkinson’s disease
Source: Sci Rep. 2019 Oct 28;9:15417. doi: 10.1038/s41598-019-51505-1 (PMC6817840; doi:10.1038/s41598-019-51505-1)
Supplement: Supplementary file 1 — Dataset1 [file 41598_2019_51505_MOESM1_ESM.docx]

Title Page

**A quick test of cognitive speed can predict development of dementia in Parkinson’s disease**

Mattis Jalakas, MD^1,2*^; Sebastian Palmqvist, MD, PhD^1,3*^; Sara Hall, MD, PhD^1,4^;; Daniel Svärd, MD ^5,6^; Olof Lindberg, PhD^1^; Joana B. Pereira , PhD^5,7^; Danielle van Westen, MD, PhD ^5,6^; Oskar Hansson MD, PhD^1,4^.

^1^ Clinical Memory Research Unit, Department of Clinical Sciences, Malmö, Lund University, Sweden

^2^ Department of Neurosurgery, Skåne University Hospital, Sweden

^3^ Department of Neurology, Skåne University Hospital, Sweden

^4^ Memory Clinic, Skåne University Hospital, Sweden

^5^ Diagnostic Radiology Lund University, Sweden

^6^ Medical Imaging and Physiology, Skåne University Hospital, Sweden

^7^ Division of Clinical Geriatrics, Department of Neurobiology, Care Sciences and Society, Karolinska Institute, Stockholm Sweden

Word Count: Article 3393 words Abstract 199 words

Running title: AQT predicts dementia in PD

Key Words: Parkinson´s, dementia, AQT, MRI, Cingulum

Financial Disclosures/Conflict of Interests: None to disclose

*Corresponding authors:

Mattis Jalakas, Clinical Memory Research Unit, Department of Clinical Sciences, Lund University, Sweden. Email: [Mattis.Jalakas@med.lu.se](mailto:Mattis.Jalakas@med.lu.se)

Or

Sebastian Palmqvist, Clinical Memory Research Unit, Department of Clinical Sciences, Lund University, Sweden. Email: [sebastian.palmqvist@med.lu.se](mailto:Mattis.Jalakas@med.lu.se)

| Supplementary table 1 | PD with MRI (n=125) |
| --- | --- |
| Baseline age, years  Years of education  UPDRS  MMSE  Wordlist delayed recall  AQT Colour Form  AQT Colour  AQT Form  LetterS fluency  Clock Drawing Test | 67 (10)  11 (5.3)  17 (12)  28 (2.6)  3.5 (2.7)  80 (43)  30 (11)  42 (18)  14 (6.0)  4.3 (0.8) |
| Standard deviations in parenthesis.  PD=106 PDD=19 | |

| Supplementary table 2. Single test risk stratification of PD dementia conversion, unadjusted. | | | |
| --- | --- | --- | --- |
| Cognitive test | HR | p-value | 95% CI |
| MMSE  AQT Colour Form  AQT Colour  AQT Form  10-world list delayed recall  Letter S fluency  Months backwards  Pentagon drawing  Clock Drawing Test | 1.2  2.0  1.4  1.8  1.6  0.6  1.1  0.42  1.6 | 0.45  0.001  0.007  0.001  0.004  0.034  0.53  0.002  0.004 | 0.77-1.7  1.3-3.0  1.1-1.7  1.4-2.3  1.2-2.2  0.45-0.97  0.83-1.4  0.24-0.73  1.2-2.3 |
| COX regression evaluating the z-scores of the cognitive tests as predictors for conversion to dementia in non-demented PD. Not adjusted. | | | |

| Supplementary table 3. Partial correlations between AQT and white-matter tracts corrected for age, sex, disease duration, brain volume and years of education | | | | |
| --- | --- | --- | --- | --- |
| Tract | Correlation dx (right) | p-value | Correlation sin (left) | p-value |
| \| CD MD  CD MK  CD FA \| \| --- \| \| CV MD  CV MK  CV FA \| \| CST MD  CST MK  CST FA \| \| UF MD  UF MK  UF FA \| \| SLF MD  SLF MK  SLA FA \| \| ILF MD  ILF MK  ILF FA  IFO MD  IFO MK  IFO FA  FX MD  FX MK  FX FA \| | **0.274**  -0.129  -0.064  **0.274**  -0.170  0.023  -0.065  -0.139  -0.005  0.192  .0.151  0.149  0.093  -0.108  0.087  **0.214**  -0.107  -0.100  **0.220**  -0.182  **-0.208**  0.036  -0.018  -0.007 | **0.007**  0.207  0.530  **0.007**  0.090  0.821  0.525  0.175  0.961  0.059  0.139  0.145  0.366  0.292  0.398  **0.035**  0.287  0.329  **0.024**  0.075  **0.041**  0.728  0.862  0.948 | **0.222**  -0.146  -0.108  0.162  **-0.216**  0.177  -0.043  -0.078  -0.003  0.139  -0.156  -0.082  0.170  -0.174  -0-054  **0.200**  -0.183  0.027  0.148  **-0.229**  0.027 | **0.030**  0.155  0.290  0.133  **0.033**  0.082  0.676  -0.448  0.978  0.175  0.128  0.423  0.096  0.089  0.602  **0.050**  0.073  0.794  0.148  **0.024**  0.790 |
| CD, Dorsal cingulum; CV, Ventral cingulum; CST, Cortico spinal tract, IFO, Inferior fronto-occipital fasciculus, ILF, inferior longitudinal fasciculus, SLF, Superior longitudinal fasciculus, UF, Uncinate fasciculus, FX fornix, represented in column dx but is not side specific | | | | |
